# Apigenin Alleviates Liver Fibrosis by Inhibiting Hepatic Stellate Cell Activation and Autophagy via TGF-*β*1/Smad3 and p38/PPAR*α* Pathways

**DOI:** 10.1155/2021/6651839

**Published:** 2021-01-28

**Authors:** Jie Ji, Qiang Yu, Weiqi Dai, Liwei Wu, Jiao Feng, Yuanyuan Zheng, Yan Li, Chuanyong Guo

**Affiliations:** Department of Gastroenterology, Shanghai Tenth People's Hospital, Tongji University School of Medicine, Shanghai 200072, China

## Abstract

**Objective:**

The aim of this study is to confirm the hepatocellular protective functions of apigenin and the molecular mechanism on liver fibrosis in mice.

**Methods:**

Carbon tetrachloride (CCl_4_) and bile duct ligature (BDL) mouse fibrosis models were used to investigate the effects of apigenin on liver fibrosis. Sixty-six male C57 mice were randomly divided into eight groups, including the vehicle group, CCl_4_ group, CCl_4_+L-apigenin (20 mg/kg) group, CCl_4_+H-apigenin (40 mg/kg) group, sham group, BDL group, BDL+L-apigenin(20 mg/kg) group, and BDL+H-apigenin(40 mg/kg) group. Serum liver enzymes (ALT and AST), proteins associated with autophagy, and indicators linked with the TGF-*β*1/Smad3 and p38/PPAR*α* pathways were detected using qRT-PCR, immunohistochemical staining, and western blotting.

**Results:**

Our findings confirmed that apigenin could decrease the levels of ALT and AST, suppress the generation of ECM, inhibit the activation of HSCs, regulate the balance of MMP2 and TIMP1, reduce the expression of autophagy-linked protein, and restrain the TGF-*β*1/Smad3 and p38/PPAR*α* pathways.

**Conclusion:**

Apigenin could alleviate liver fibrosis by inhibiting hepatic stellate cell activation and autophagy via TGF-*β*1/Smad3 and p38/PPAR*α* pathways.

## 1. Introduction

Liver fibrosis is a chronic pathological change caused by a variety of reasons, such as chronic infection by hepatotropic viruses, excess alcohol consumption, nonalcoholic fatty liver disease, autoimmune liver diseases, and hereditary disease, and is a necessary stage for the development of many liver diseases to liver cirrhosis and even liver cancer [1, 2]. Liver fibrosis is a wound healing response characterized by excessive deposition of extracellular matrix (ECM). The possible treatments for liver fibrosis including curing the primary disease, reducing inflammation and immune response, inhibiting stellate cell activation, and increasing the degradation of scar matrix had been generally accepted [3, 4]. Although liver transplantation is the most efficient therapy, there are great limitations because of huge cost of treatment and shortage of liver donor available for transplantation [1, 5, 6]. Therefore, revealing the molecular mechanism of liver fibrosis and finding key drug targets are an important issue that needs to be solved urgently.

The progression of fibrosis is a complex process which involves nonparenchymal hepatocytes, parenchymal hepatocytes, and infiltrating immune cells. The activation of inflammation mediators and profibrotic genes caused by cell death in both nonparenchymal and infiltrating immune cells thereby trigger the fibrosis process. Hepatic stellate cells (HSCs) are the most powerful fibrogenic effector cells and are also considered as the initial process during liver fibrosis [4, 7–10]. The activation of HSCs by several cellular events including immune/inflammatory injury as well as molecular regulation especially transforming growth factor-*β*1 (TGF-*β*1) will contribute to the excessive accumulation of ECM which promotes liver fibrosis [11]. It had been reported that suppressing the activation HSCs and expression of TGF-*β*1 could reduce the levels of myofibroblast markers, increase the ratio of MMPs/TIMPs, and decrease Smad2/Smad3 associated collagen production which further attenuated liver fibrosis [11–15].

Autophagy is a self-selective mode of cell death, which can remove necrotic cells to maintain organ homeostasis [12]. Results have shown that autophagy could provide energy for the activation of HSCs by stimulating the metabolism of lipid droplets [16]. At the same time, many literatures have confirmed that inhibiting the autophagy of HSCs can play a positive role in liver protection [17–20]. So, inhibition of autophagy which could significantly reduce activation of HSCs can attenuate liver fibrosis [21, 22].

Apigenin is a kind of dietary flavonoid extracted mainly from celery, parsley, thyme, chamomile, and onions [23]. Recently, apigenin has reported many pharmacological effects including anticancer [24–28], anti-inflammation [29–32], antifibrosis [33–36], and so on. Zhang et al. confirmed apigenin could downregulate the miR34a expression to suppress mouse peritoneal fibrosis [35]. Jiao et al. demonstrated that apigenin could inhibit fibroblast proliferation and reduce epidural fibrosis by suppressing the Wnt3a/*β*-catenin signaling pathway [34]. However, whether apigenin has the antihepatic fibrosis effect and the specific molecular mechanism of this effect are still unclear and need to be explored.

Carbon tetrachloride (CCl_4_) and bile duct ligature (BDL) mouse models are extremely practical models to investigate the underlying molecular mechanisms of liver fibrosis, which have been widely applied to the establishment of liver fibrosis [15, 37]. Therefore, this study is aimed at exploring the antihepatic fibrosis effect and the specific molecular mechanism of apigenin using the CCl_4_ and BDL models. We hypothesized that apigenin could alleviate liver fibrosis by inhibiting hepatic stellate cell activation and autophagy via TGF-*β*1/Smad3 and p38 MAPK/PPAR*α* pathways.

## 2. Materials and Methods

### 2.1. Drugs and Reagents

Apigenin (HPLC ≥ 98% CAS:520-36-5) was purchased from Shanghai Yuanye Bio-Technology Co., Ltd. (Shanghai, China). When used, it is dissolved into dimethyl sulfoxide (DMSO) at 2 mg/ml and 4 mg/ml concentrations. Carbon tetrachloride (CCl_4_) was purchased from China Sinopharm International Corporation (Shanghai, China). Alanine aminotransferase (ALT) and aspartate aminotransferase (AST) were tested by microplate test kits purchased from Nanjing Jiancheng Bioengineering Institute (Nanjing, China). Quantitative real-time (qRT) PCR kits were purchased from TaKaRa (Dalian, China). The primers were obtained from Generay (Shanghai, China). Detailed information of the primary antibodies used in this study are listed in Table 1. Dulbecco's Modified Eagle Medium (DMEM) and foetal bovine serum (FBS) were purchased from HyClone (GE Healthcare). Apigenin was dissolved in DMSO (<0.1% [*v*/*v*]) for in vitro treatment.

### 2.2. Cell Culture and CCK8 Assay

The human immortal LX2 cell line was cultured in high glucose DMEM with 10% FBS, 100 U/mL of penicillin, and 100 g/mL of streptomycin. The apparent logarithmic phase cells were seeded in 96-well plates for 48 hours, then apigenin was added at concentrations of 10, 20, 30, 40, 50, 60, 70, or 80 *μ*M for 24 hours, and the cytotoxicity analysis was performed. Cell viability was then measured with the CCK8 assay according to the manufacturer's protocol. All the experiments were performed in triplicate.

### 2.3. BrdU Assay

Proliferation of the cells was evaluated using the BrdU Cell Proliferation ELISA Kit (ab126556, Abcam, Cambridge, MA) according to the manufacturer's instruction. Briefly, cells were cultured in 96-well plates and exposed to apigenin (20, 40, and 60 *μ*M) for 24 hours. Subsequently, 10 *μ*M BrdU was added to each well, and samples were incubated for 12 h at 37°C. BrdU signaling was determined by measuring the absorbance at 450 nm.

### 2.4. Animals

66 six-week-old male C57 mice (22-26 g) were obtained from Shanghai SLAC Laboratory Animal (Shanghai, China) and housed in a standard animal laboratory with free access to food and water. All experimental procedures involving mice were approved by the Animal Care and Use Committee of Shanghai Tongji University. Handling and care of mice conformed to the National Institutes of Health Guidelines.

### 2.5. Establishment of Mouse Liver Fibrosis Models

We established two different mouse liver fibrosis models. To create the CCl_4_-induced liver fibrosis model, mice were injected with 10% CCl4 (1.0 mL/kg, diluted in peanut oil) intraperitoneally three times a week for 8 weeks. In the bile duct ligation- (BDL-) induced liver fibrosis model, all mice were fasted for 12 h and anesthetized intraperitoneally by 1.25% pentobarbital sodium salt (40 mg/kg). After opening the abdomen via the linea alba, the bile duct was exposed and isolated over a certain length. Two surgical knots were tied in the isolated bile duct, which was then cut between the knots. The abdomen was then closed.

### 2.6. Experimental Design

#### 2.6.1. Preliminary Study

In order to verify whether the apigenin dose (20 mg/kg and 40 mg/kg) could cause damage to the structure and function of the liver and other internal organs, we designed a preliminary experiment. The eighteen mice were randomly divided into the following 3 groups.

1. Normal control (NC) (*n* = 6): no treatment
2. Vehicle group (*n* = 6): mice were injected intraperitoneally with DMSO three times a week
3. API (40 mg/kg) group (*n* = 6): apigenin (40 mg/kg) was given to mice by intragastric administration three times a week.

#### 2.6.2. Formal Experiment

In the CCl_4_-induced liver fibrosis model, 24 mice were randomly divided into the following 4 groups.

1. Vehicle group (*n* = 6): mice were injected intraperitoneally with DMSO three times a week for 8 weeks
2. CCl_4_ group (*n* = 6): mice were injected with CCl_4_ intraperitoneally three times a week for 8 weeks
3. CCl_4_+L-API group (*n* = 6): mice were injected with CCl_4_ intraperitoneally and gavaged with 20 mg/kg apigenin three times a week for 8 weeks
4. CCl_4_+H-API group (*n* = 6): mice were injected with CCl_4_ intraperitoneally and gavaged with 40 mg/kg apigenin three times a week for 8 weeks

In the BDL-induced liver fibrosis model, 24 mice were randomly divided into the following 4 groups.

1. Sham group (*n* = 6): all mice underwent laparotomy without BDL
2. BDL group (*n* = 6): all mice underwent BDL surgery
3. BDL+L-API group (*n* = 6): all mice were gavaged with 20 mg/kg apigenin once a day for 14 days after BDL
4. BDL+H-API group (*n* = 6): all mice were gavaged with 40 mg/kg apigenin once a day for 14 days after BDL

Vehicle and sham groups were used as controls in both models. At the end of the experiment, blood samples and liver tissues were collected with diethyl ether anesthesia. Serum was acquired by centrifugation (4,500 rpm, 4°C, 10 min) and kept at −80°C. Liver tissues were stored at −80°C.

### 2.7. Serum Biochemical Analysis

The blood sample collected from the mouse orbit was placed at 4°C for 5 hours. And then, the serum sample was separated from the blood by centrifuging at 4,600 × g at 4°C for 10 minutes. Serum levels of ALT and AST were detected by microplate test kits.

### 2.8. Histopathology

A part of the fresh left liver lobe was excised and then fixed in 4% paraformaldehyde for 24 h. The tissues were dehydrated with ethanol and embedded in paraffin. Next, the liver tissues were cut into 3 *μ*m thick sections and stained with hematoxylin and eosin (H&E) to determine the severity of injury.

### 2.9. Reverse Transcription PCR (RT-PCR) and Quantitative Real-Time PCR (qRT-PCR)

The total RNA was extracted from 100 mg liver tissue by TRIzol (Thermo Fisher Scientific, Waltham, MA, USA). Then, the purified RNA was reverse-transcribed into cDNA. The levels of mRNA were determined by SYBR Premix EX Taq through a 7900HT fast PCR system (Applied Biosystems, Foster City, CA, USA). The primers used for qRT-PCR are listed in Table 2.

### 2.10. Immunohistochemistry (IHC)

Paraffin sections were baked in a 60°C oven for 1 hour and then dewaxed and rehydrated. Antigen was placed into a citrate buffer, which was then heated to 95°C for 10 minutes and cooled to room temperature. Next, the sections were covered in 3% hydrogen peroxide for 20 minutes to block endogenous peroxidase activity, and then 5% BSA was added to block nonspecific binding for 15 minutes (both at room temperature). Slices were then incubated overnight at 4°C with the following antibodies: anti-IL-1*β*, anti-*α*-SMA, anti-Col, anti-LC3, anti-Beclin-1, anti-p62-, anti-TGF-*β*1-, anti-p-Smad3-, anti-p-38-, and anti-PPAR*α* (all 1 : 200). Then, the primary antibodies in the liver sections were incubated with secondary antibodies using a diaminobenzidine (DAB) kit. Final evaluations were performed with Image-Pro Plus software 6.0 to calculate the mean of integrated optical densities (MIOD = sum IOD/sum area) of the positive staining area.

### 2.11. Western Blotting

Firstly, liver tissues were ground (100 mg) into powder in liquid nitrogen, and then the powder was homogenized in RIPA lysis containing phenylmethanesulfonyl fluoride (PMSF) and protease inhibitors (PI). The protein concentrations were detected using the bicinchoninic acid method before being mixed with a 6x loading buffer and boiled at 100°C for 10 minutes. Secondly, protein samples were electrophoresed by 10% or 12.5% SDS-PAGE and transferred onto polyvinylidene fluoride or nitrocellulose membranes. Next, membranes were blocked with 5% skimmed milk for at least 1 hour and subsequently incubated overnight at 4°C with the primary antibodies (Table 1). Thirdly, the membranes were incubated with anti-rabbit or anti-mouse secondary antibodies after washing thrice with PBST (1% Tween diluted in PBS). Finally, the expression of protein was measured by an Odyssey two-color infrared laser imaging system (LI-COR Biosciences, Lincoln, NE, USA).

### 2.12. Statistical Analysis

Experimental data which was repeated at least three times was presented as mean ± SD (*n* = 6; ^∗^*P* < 0.05 for CCl_4_ (BDL) vs. control; ^#^*P* < 0.05 for CCl_4_ (BDL)+API (20 mg/kg) vs. CCl_4_ (BDL); ^+^*P* < 0.05 for CCl_4_ (BDL)+API (40 mg/kg) vs. CCl_4_ (BDL); ^!^*P* < 0.05 for CCl_4_ (BDL)+API (40 mg/kg) vs. CCl_4_ (BDL)+API(20 mg/kg)). One-way ANOVA using the Student–Newman–Keuls method was used to compare statistical differences among three or four groups using SPSS version 20.0 software (IBM, Armonk, NY, USA). *P* < 0.05 was regarded as statistically significant.

## 3. Result

### 3.1. Effects of Apigenin on Liver and LX2 Cells

**Figure 1:**
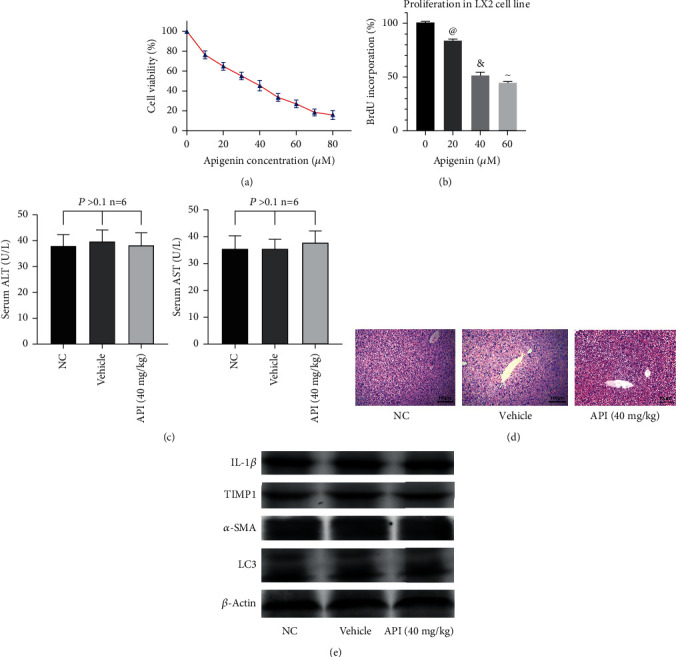
Effects of apigenin on liver and LX2 cells. Notes: (a) the CCK8 assay was used to determine the effects of apigenin on the viability of LX2 cells. (b) Cells were treated with the indicated concentrations of apigenin for 24 hours, and the degree of apigenin to inhibit cell proliferation was measured using BrdU Cell Proliferation ELISA Kit (^@^*P* < 0.05 for 20 *μ*M apigenin vs. 0 *μ*M apigenin; ^&^*P* < 0.05 for 40 *μ*M apigenin vs. 20 *μ*M apigenin; ^~^*P* < 0.05 for 60 *μ*M apigenin vs. 40 *μ*M apigenin). (c) The levels of serum ALT and AST are presented as mean ± SD. One-way ANOVA indicated that there was no significant difference among the three groups (*n* = 6; *P* > 0.1). (d) Representative H&E-stained hepatic sections were examined under light microscopy and imaged at a 200x magnification. (e) Western blot analysis of IL-1*β*, TIMP1, *α*-SMA, and LC3 protein levels.

The human immortal HSC cell line (LX2 cells) was used in this study to investigate the effect of apigenin on HSCs. The CCK8 assay was used to measure the toxicity of apigenin in LX2 cells (Figure 1(a)). Apigenin decreased the viability of LX2 cells in a dose-dependent manner, and the half-maximal inhibitory concentration (IC50) was 28.80 *μ*M. At the same time, the BrdU incorporation assay was performed to explore the effect of apigenin on cell proliferation. As shown in Figure 1(b), apigenin could reduce the proportion of proliferating cells in a dose-dependent manner. Besides, in the preliminary experiment, 12 mice were injected with vehicle (DMSO) or gavaged with 40 mg/kg apigenin to explore security of drug and solvent used in this study. As shown in Figure 1(c), there was no hepatocellular injury or structural damage compared with the NC group. The results of ALT, AST, and western blotting shown in Figures 1(b) and 1(d) could also verify no statistically significant differences between the vehicle, apigenin, and NC groups. So, we got the conclusion that the apigenin could inhibit proliferation and decrease the viability of LX2 cells, but has no harmful effects on the liver tissues.

### 3.2. Apigenin Protects the Liver against Fibrosis Induced by CCl_4_ and BDL in Mice

**Figure 2:**
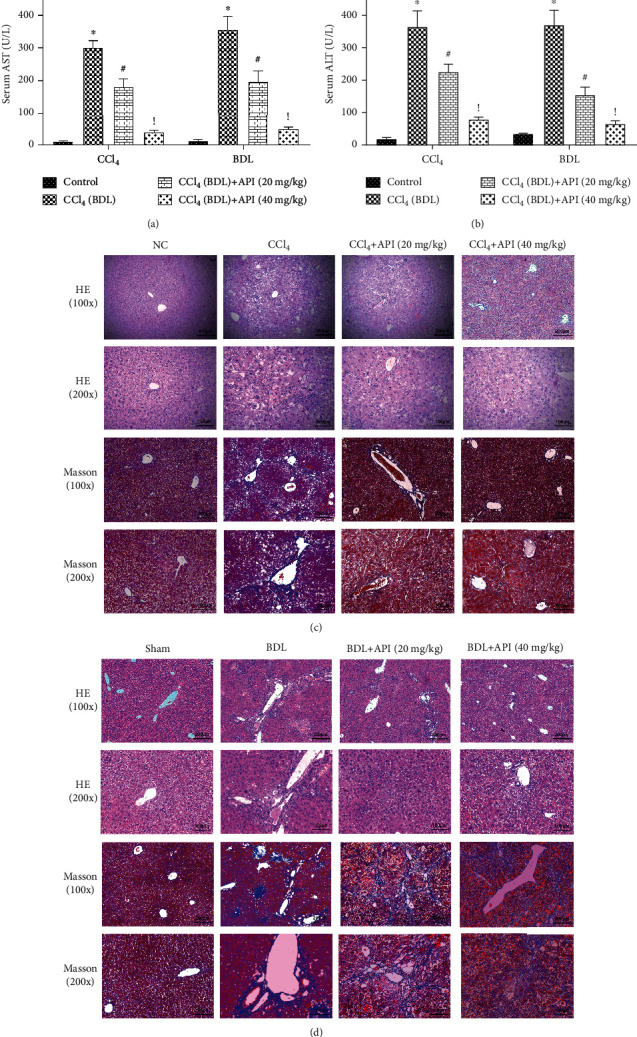
Apigenin protects the liver against fibrosis induced by CCl_4_ and BDL in mice. Notes: (a, b) the levels of serum ALT and AST are presented as mean ± SD (*n* = 6; ^∗^*P* < 0.05 for CCl_4_ (BDL) group vs. control group; ^#^*P* < 0.05 for CCl_4_ (BDL)+apigenin (20 mg/kg) group vs. CCl_4_ (BDL) group; ^!^*P* < 0.05 for CCl_4_ (BDL)+apigenin (40 mg/kg) group vs. CCl_4_ (BDL)+apigenin (20 mg/kg) group). (c, d) Representative H&E- and Masson-stained hepatic sections were examined under light microscopy and imaged at 200x and 100x magnifications.

The levels of serum ALT and AST are important indicators of liver parenchymal damage. So, we detected the levels of ALT and AST in the serum to explore the extent of liver parenchymal damage in both fibrosis models. We could see it clearly from the Figures 2(a) and 2(b) that ALT and AST elevated dramatically in model groups compared with vehicle and sham groups. However, we also noticed apigenin groups could reverse the increase induced by CCl_4_ and BDL surgery in a dose-dependent manner. Next, HE and Masson staining were used to evaluate the pathological changes of liver tissues. HE staining showed that the morphology and structure of mouse liver cells in the control groups were normal, with normal arrangement, normal hepatic lobules and portal area, and no inflammatory cell exudation. Compared with the control groups, the disordered arrangement of liver cells, the damaged normal structure, the exudation of many inflammatory cells, and the proliferation of collagen fibers were significantly observed in the CCl_4_ and BDL groups. When apigenin was given at the same time, the disordered arrangement of liver cells was significantly reduced, the structure of portal area was almost normalized, fibrous tissue hyperplasia and inflammatory cell infiltration were significantly decreased, and the morphological structure was close to normal liver tissue (Figures 2(c) and 2(d)). The results of Masson staining could further confirm the protective effect on liver fibrosis of apigenin. The above results showed that apigenin had an obviously protective effect on CCl_4_- and BDL-induced liver fibrosis in mice.

### 3.3. Apigenin Restrained the Activation of HSC and Regulated the Balance of TIMP1 and MMP2

**Figure 3:**
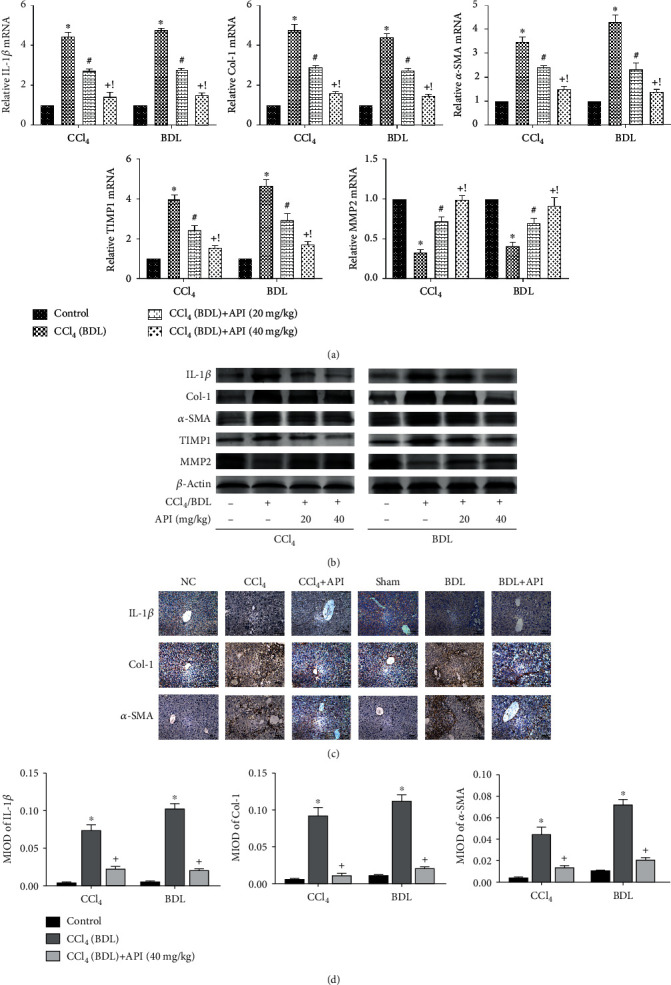
Apigenin restrained the activation of HSC and regulated the balance of TIMP1 and MMP2. Notes: (a) relative IL-1*β*, Col-1, *α*-SMA, TIMP1, and MMP2 mRNA levels were determined by qRT-PCR. (b) Western blot analysis of IL-1*β*, Col-1, *α*-SMA, TIMP1, and MMP2 protein levels. (c) IL-1*β*, Col-1, and *α*-SMA protein expressions in liver tissues are shown by immunohistochemical staining (200x). (d) Final evaluations were made using Image-Pro Plus 6.0 software to calculate the MIOD of the positive staining area. Data are presented as mean ± SD (*n* = 6; ^∗^*P* < 0.05 for CCl_4_ (BDL) group vs. control group; ^#^*P* < 0.05 for CCl_4_ (BDL)+apigenin (20 mg/kg) group vs. CCl_4_ (BDL) group; ^+^*P* < 0.05 for CCl_4_ (BDL)+apigenin (40 mg/kg) group vs. CCl_4_ (BDL) group; ^!^*P* < 0.05 for CCl_4_ (BDL)+apigenin (40 mg/kg) group vs. CCl_4_ (BDL)+apigenin (20 mg/kg) group). Abbreviation: MIOD: mean of integrate optical density.

*α*-SMA was an important indicator of HSC activation, and collagen 1 was the main component of ECM, which were often used as important indicators to test the degree of liver fibrosis. In order to further prove the effect of apigenin on mouse liver fibrosis, mRNA and protein expression of collagen 1, *α*-SMA, and IL-1*β* in the mouse liver tissues were measured by real-time PCR, western blotting, and IHC. The results showed that compared with the vehicle or sham control groups, mRNA and protein expression of collagen 1, *α*-SMA, and IL-1*β* in the model group were significantly increased, but their expressions were decreased after apigenin treatment (Figures 3(a)–3(c)). The synthesis and degradation of hepatic ECM are regulated by matrix metalloproteinases (MMPs) and matrix metalloproteinase inhibitors (TIMPs). Injury factors can lead to the activation of HSC, resulting in the imbalance of MMPS/TIMPs; therefore, we measured the levels of TIMP1 and MMP2 in liver tissues. The results indicated that the expression of TIMP1 increased obviously in the CCl_4_ and BDL groups, and this trend could be inhibited by apigenin treatment. On the contrary, MMP2 decreased in the fibrosis model groups but increased in the apigenin groups. In general, the above experimental results showed that apigenin could restrain the activation of HSC and regulated the balance of TIMP1 and MMP2 to relieve liver fibrosis in mice.

### 3.4. Apigenin Alleviated Autophagy during Liver Fibrosis

**Figure 4:**
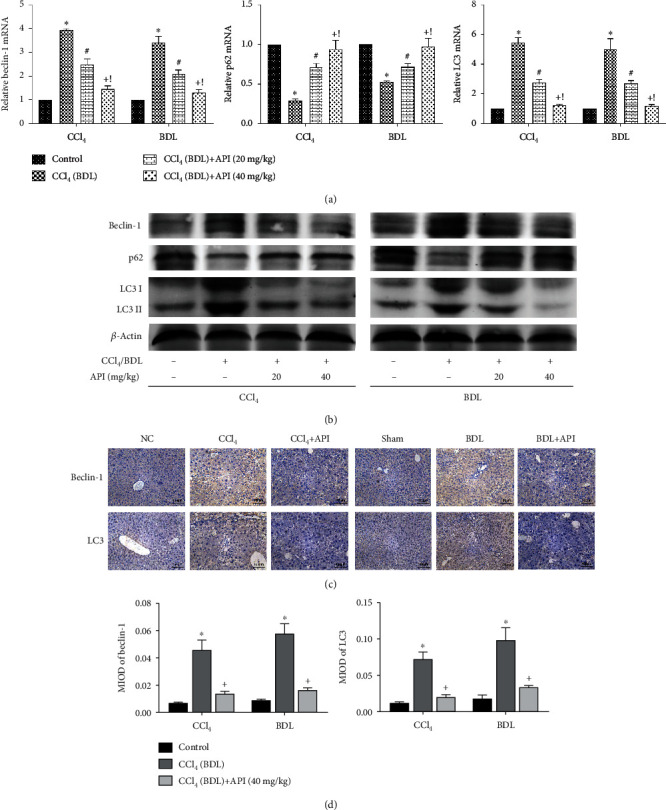
Apigenin alleviated autophagy during liver fibrosis. Notes: (a) relative Beclin-1, p62, and LC3 mRNA levels were determined by qRT-PCR. (b) Western blot analysis of Beclin-1, p62, and LC3. (c) Beclin-1 and LC3 protein expressions in liver tissues are shown by immunohistochemical staining (200x). (d) Final evaluations were made using Image-Pro Plus 6.0 software to calculate the MIOD of the positive staining area. Data are presented as mean ± SD (*n* = 6; ^∗^*P* < 0.05 for CCl_4_ (BDL) group vs. control group; ^#^*P* < 0.05 for CCl_4_ (BDL)+apigenin (20 mg/kg) group vs. CCl_4_ (BDL) group; ^+^*P* < 0.05 for CCl_4_ (BDL)+apigenin (40 mg/kg) group vs. CCl_4_ (BDL) group; ^!^*P* < 0.05 for CCl_4_ (BDL)+apigenin (40 mg/kg) group vs. CCl_4_ (BDL)+apigenin (20 mg/kg) group). Abbreviation: MIOD: mean of integrate optical density.

Beclin-1, LC3, and p62, which are autophagy signature proteins, were analyzed by qRT-PCR, IHC, and western blotting to explore the protective effect of apigenin. As demonstrated in Figures 4(a)–4(d) and S1, the expressions of Beclin-1 and LC3II/LC3I augmented obviously, while p62 decreased drastically, in the CCl_4_ and BDL groups. However, apigenin groups could ameliorate these changes in a dose-dependent manner. The above results suggested that apigenin could alleviate autophagy during liver fibrosis.

### 3.5. Apigenin Could Relieve Hepatic Fibrosis Induced by CCl_4_ and BDL via Downregulating TGF-*β*1/Smad3 and p38/PPAR*α* Pathways

**Figure 5:**
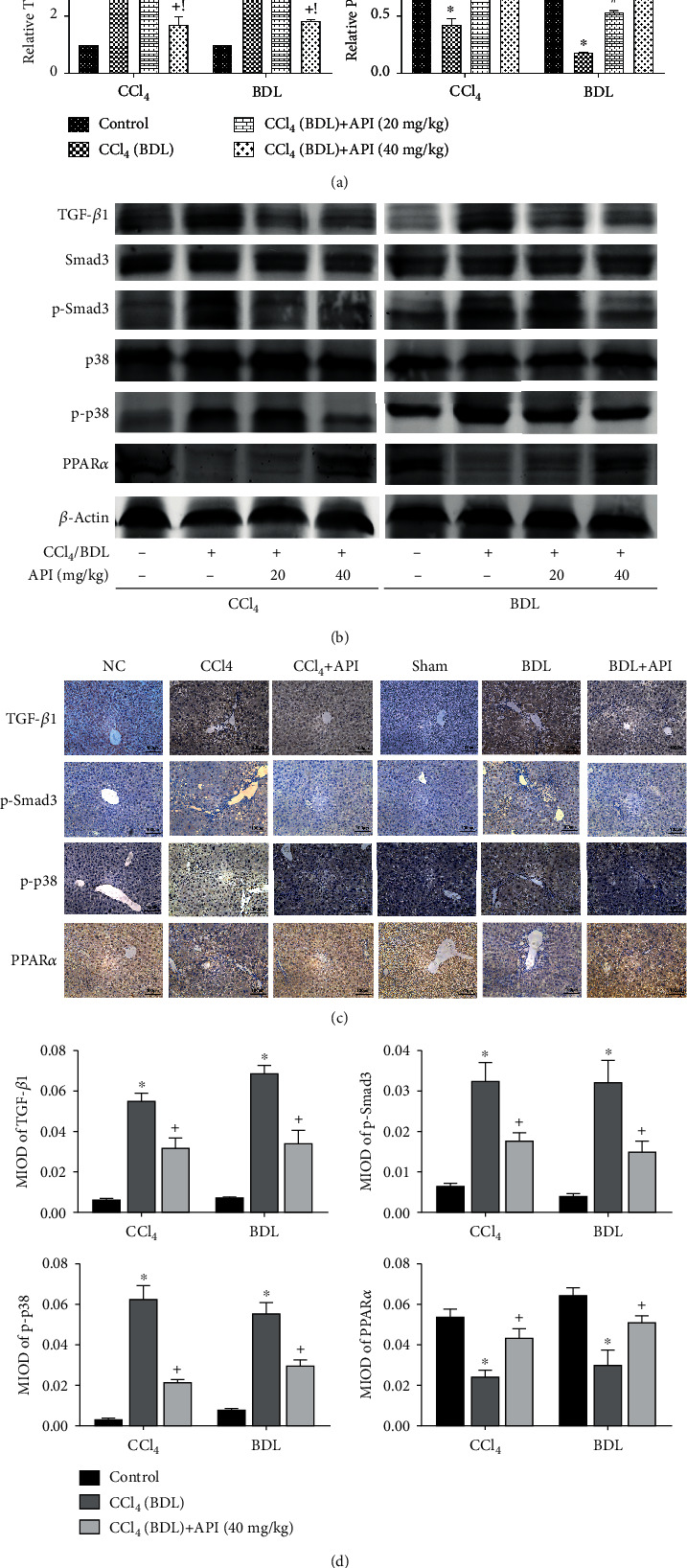
Apigenin could relieve hepatic fibrosis induced by CCl_4_ and BDL via downregulating TGF-*β*1/Smad3 and p38/PPAR*α* pathways. Notes: (a) relative TGF-*β*1 and PPAR*α* mRNA levels were determined by qRT-PCR. (b) Western blot analysis of TGF-*β*1, Smad3, p-Smad3, p38, p-p38, and PPAR*α*. (c) TGF-*β*1, p-Smad3, p-p38, and PPAR*α* protein expressions in liver tissues are shown by immunohistochemical staining (200x). (d) Final evaluations were made using Image-Pro Plus 6.0 software to calculate the MIOD of the positive staining area. Data are presented as mean ± SD (*n* = 6; ^∗^*P* < 0.05 for CCl_4_ (BDL) group vs. control group; ^#^*P* < 0.05 for CCl_4_ (BDL)+apigenin (20 mg/kg) group vs. CCl_4_ (BDL) group; ^+^*P* < 0.05 for CCl_4_ (BDL)+apigenin (40 mg/kg) group vs. CCl_4_ (BDL) group; ^!^*P* < 0.05 for CCl_4_ (BDL)+apigenin (40 mg/kg) group vs. CCl_4_ (BDL)+apigenin (20 mg/kg) group). Abbreviation: MIOD: mean of integrate optical density.

TGF-*β*1 is a pluripotent cytokine that is involved in inflammatory infiltration, cell growth, apoptosis, differentiation, and other processes in fibrosis. The Smad protein family is the downstream molecule of TGF-*β*1. Therefore, we evaluated the expressions of the TGF-*β*1/Smad3 pathway. The results of qRT-PCR, IHC, and western blotting in Figures 5(a)–5(d) illustrated that CCl_4_ and BDL surgery could significantly active the TGF-*β*1/Smad3 pathway, but apigenin treatment could reverse this activation. It means that the protective effects of apigenin were associated with restraining the TGF-*β*1/Smad3 pathway. Next, we measured the levels of p38 and PPAR*α* which was also a downstream molecule of TGF-*β*1. In our results, we found that liver fibrosis induced by CCl_4_ and BDL surgery could lead to phosphorylation of p38, which further inhibited PPAR*α*. In apigenin treatment groups, p-p38 was dramatically downregulated and PPAR*α* increased obviously. Therefore, we can draw the conclusion that apigenin could relieve hepatic fibrosis induced by CCl4 and BDL via downregulating the TGF-*β*1/Smad3 and p38/PPAR*α* pathways.

## 4. Discussion

Liver fibrosis is a chronic wounding-healing response with a long-time liver injury [4, 38]. Although there are little symptoms at the beginning of liver fibrosis, the risk of mortality increases significantly once liver fibrosis progresses to cirrhosis and even hepatocellular carcinoma [2]. More than 30,000 deaths per year caused by cirrhosis and 1,000 deaths per year occurred related to liver cancer in the United States are enough to warn us that halting and reversing the progression of fibrosis is currently an effective way to reduce mortality rather than only relying on highly limited liver transplants [39].

Apigenin is a kind of dietary flavonoid extracted mainly from celery, parsley, thyme, chamomile, and onions [23]. It had been reported that apigenin is of great effect in antifibrosis [33–36] and liver protection [26, 40–42]. Mirzoeva et al. demonstrated that apigenin could reduce TGF-*β*-induced VEGF production and suppress prostate carcinogenesis by regulating the Smad2/3 and Src/Fak/Akt pathways. Apigenin is also reported to inhibit metastasis and angiogenesis by the p38 MAPK pathway [43].These indicate that apigenin may become an efficient drug to prevent liver fibrosis, and the molecular mechanism might be closely related to the TGF-*β* and p38 MAPK pathway. Therefore, in our study, CCl_4_- and BDL-induced liver fibrosis models are used to explore the effects of apigenin and the specific molecular mechanism. Our results of HE and Masson staining confirmed that apigenin could improve liver fibrosis in a dose-dependent manner.

The first step to try to stop and reverse liver fibrosis is to explore the molecular mechanisms of this disease. The formation of liver fibrosis is a complex pathophysiological process involving many cells, molecules, and signaling pathways. The accumulation of ECM is regarded as the important character, and the activation of HSCs is considered as the initial process of liver fibrosis [2, 7, 10]. HSCs are one of the mesenchymal cells which account for one-third of the nonparenchymal cells in the liver and 15% of the total number of liver cells [11]. In normal conditions, HSC is at a quiescent condition and could store vitamin A and triglycerides in the cytoplasm [44]. However, when the liver suffers from acute or chronic injury, HSCs are activated and differentiated into myofibroblasts, which have a strong ability of proliferation, migration, and secretion. Activated HSCs are the main cells to produce ECM, and a large amount of ECM is continuously deposited in the Disse space. In addition, the main components of ECM also change from type IV collagen to type I collagen [45], resulting in the increase of density and hardness of ECM, and accumulated ECM also becomes the liver fibrosis tissue microenvironment containing *α*-SMA, TGF-*β*1, chemokines such as PDGF, hepatocyte growth factor (HGF), fibroblast growth factor (FGF), epidermal growth factor (EGF), and VEGF [46]. The synthesis and degradation of liver ECM is regulated by the combination of matrix metalloproteinases (MMPs) and tissue matrix metalloproteinase inhibitors (TIMPs). Under normal conditions, MMPs and TIMPs can be synthesized by hepatocytes and various mesenchymal cells and play a key role in maintaining the dynamic balance between ECM synthesis and degradation in normal liver tissues through complex regulatory mechanisms [12, 13, 47]. In our study, we explored the function of apigenin in the activation of HSCs and the levels of ECM. Our results illustrated that apigenin could suppress the activation of HSCs and decrease ECM by increasing the ratio of MMP2/TIMP1.

TGF-*β* is generally considered to be the strongest fibrogenic factor. The activation of the TGF-*β*1/Smad3 signaling pathway plays an important role in liver fibrosis [48]. Smad3 is phosphorylated into p-Smad3 which could promote the transcription of type 1 and type 3 collagen after the activation of TGF-*β*1 [49]. In addition, TGF-*β* can also activate the p38 MAPK signaling pathway to promote the transcription of collagen which is the main ingredient of ECM [50]. Besides, large amounts of literature have confirmed that inhibiting the TGF-*β*1/Smad pathway could efficiently reduce the injury of liver fibrosis [4, 15, 47, 51–53]. In our study, we proved that TGF-*β*1 and Smad3 expressed much more in fibrosis model groups than in control groups, and at the same time, apigenin groups obviously reduced the expression of TGF-*β*1, Smad3, and the other related proteins. Thus, we concluded that the protective effect of apigenin was closely related to the inhibition of the TGF-*β*1/Smad3 pathway.

p38 MAPK belongs to the family of MAPKs that affects a variety of intracellular responses including cell-cycle regulation, inflammation, cell death, and tumorigenesis [54]. p38 MAPK could be phosphorylated by many extracellular stimulants through a classic MAPK pathway, and phosphorylated p38 (p-p38) could further regulate many substrates that include transcription factors, peroxisome proliferator-activated receptors (PPARs), and so on [54, 55]. The study of Liu et al. demonstrated that p38 MAPK activated by TGF-*β*1 could exert a positive effect on liver fibrosis [12]. In addition, Lu et al. illustrated that the inhibition of p-p38 MAPK could increase the expression of PPAR*α* to protect liver from concanavalin A-induced injury [55]. PPARs which belong to the subfamily of the nuclear receptor superfamily containing PPAR*α*, PPAR *β*/*δ*, and PPAR*γ* have many biological functions such as liver protection, antitumor, antiasthma, antidiabetes, and antineuropathic pain [56–62]. It was reported that PPAR*α* could reverse fibrosis by reducing lipid peroxides and inhibiting the activation of HSCs and Kupffer cells (KCs) [63, 64]. So, in our study, we detected the expressions of p-p38 and PPAR*α* and proved that apigenin could inhibit the phosphorylation of p38 which further increased PPAR*α* to protect the liver from fibrosis.

Autophagy is a self-selective mode of cell death which contributes a lot to the basic liver functions [65]. Hernandez-Gea and Friedman demonstrated that autophagy could provide energy for the activation of HSCs by stimulating metabolism of lipid droplets [16]. However, inappropriate autophagy activity may aggravate damage in hepatic injury such as liver fibrosis [66]. The conclusion of Li et al. proved that suppressing autophagy could alleviate liver fibrosis [52]. Autophagy is closely related to the TGF-*β*1/Smad3 pathway, which could increase the expression of Bechin1 and LC3 and decrease the generation of p62 [67]. In addition, the inhibition of autophagy via the p38/PPAR*α* pathway could exert positive effects in liver injury [55]. Our current results confirmed that apigenin could ameliorate liver fibrosis by inhibiting autophagy via the TGF-*β*1/Smad3 and p38/PPAR*α* pathways.

**Figure 6:**
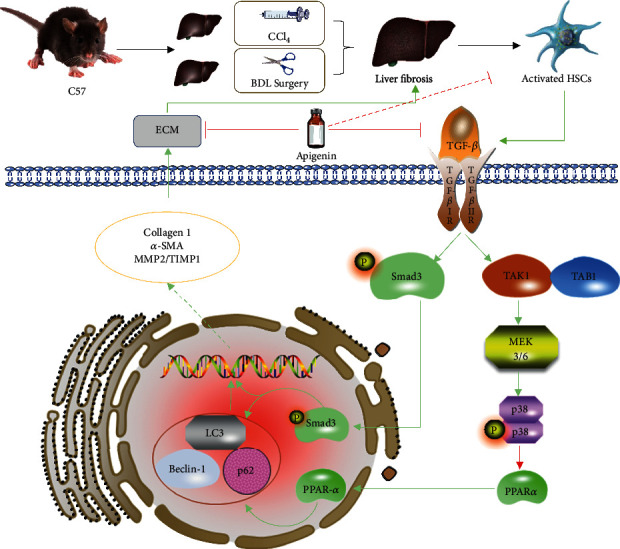
Probable mechanisms of apigenin against liver fibrosis. Notes: apigenin inhibits the production of TGF-*β*1 in HSCs. The decrease of TGF-*β*1 results in the reduced activation of HSCs and downregulation of the TGF-*β*1/Smad3 and p38/PPAR*α* signaling pathway, including decreased ECM production and inhibition of autophagy. Abbreviations: BDL: bile duct ligation; CCl_4_: carbon tetrachloride; ECM: extracellular matrix; HSCs: hepatic stellate cells.

In general, our study illustrated the liver-protective effect of apigenin in CCl_4_- and BDL-induced liver fibrosis models. Apigenin could inhibit the activation of HSCs which promote the accumulation of ECM and the secretion of many fibrogenic factors such as *α*-SMA and collagen 1. In addition, the TGF-*β*1/Smad3 and p38/PPAR*α* pathways are proven to be the main signaling pathways through which apigenin exerts its function (Figure 6). Therefore, apigenin may be a new clinical option for the treatment of fibrosis; however, more drug safety and clinical trials need to be accomplished before clinical applications.

## 5. Conclusion

Our study illustrated the liver-protective effect of apigenin in CCl_4_- and BDL-induced liver fibrosis models. Inhibiting the TGF-*β*1/Smad3 and p38/PPAR*α* pathways, reducing autophagy, and decreasing ECM formation are the major mechanism of the antifibrotic effects of apigenin.

## Figures and Tables

**Table 1:** The primary antibodies used for western blotting and immunohistochemistry in the study.

Antibody	Species	Targeted species	Supplier	Catalogue number
*β*-Actin	M	H, M, R	CST	3700
IL-1*β*	Rbt	M	CST	12507
*α*-SMA	M	H, M, R	Abcam	ab7817
Collagen 1	Rbt	H, M, R	Abcam	ab34710
MMP2	Rbt	H, M, R	PT	10373-2-AP
TIMP1	Rbt	H, M, R	PT	10753-1-AP
p62	Rbt	H, M, R	PT	55274-1-AP
LC3	Rbt	H, M, R	PT	14600-1-AP
Beclin-1	Rbt	H, M, R	PT	11306-1-AP
TGF-*β*1	Rbt	H, M, R	PT	21898-1-AP
Smad3	Rbt	H, M, R	Abcam	ab40854
p-Smad3	Rbt	H, M	Abcam	ab52903
p38 MAPK	Rbt	H, M, R	Zenbio	200782
p-p38 MAPK	Rbt	H, M, R	CST	4511
PPAR*α*	Rbt	H, M, R	PT	15540-1-AP

Abbreviations: H: human; M: mouse; Rbt: rabbit; R: rat; CST: Cell Signaling Technology (Danvers, MA, USA); PT: Proteintech (Chicago, IL, USA).

**Table 2:** Oligonucleotide sequences of primers used for qRT-PCR.

Gene name	Forward (5′-3′)	Reverse (5′-3′)
*β*-Actin	GTGACGTTGACATCCGTAAAGA	GCCGGACTCATCGTACTCC
IL-1*β*	GAAATGCCACCTTTTGACAGTG	TGGATGCTCTCATCAGGACAG
Collagen 1	CAATGGCACGGCTGTGTGCG	AGCACTCGCCCTCCCGTCTT
*α*-SMA	CCCAGACATCAGGGAGTAATGG	TCTATCGGATACTTCAGCGTCA
MMP2	GGACAAGTGGTCCGCGTAAA	CCGACCGTTGAACAGGAAGG
TIMP1	CGAGACCACCTTATACCAGCG	ATGACTGGGGTGTAGGCGTA
p62	GAGGCACCCCGAAACATGG	ACTTATAGCGAGTTCCCACCA
LC3	TTATAGAGCGATACAAGGGGGAG	CGCCGTCTGATTATCTTGATGAG
Beclin-1	ATGGAGGGGTCTAAGGCGTC	TGGGCTGTGGTAAGTAATGGA
TGF-*β*1	CCACCTGCAAGACCATCGAC	CTGGCGAGCCTTAGTTTGGAC
PPAR*α*	AACATCGAGTGTCGAATATGTGG	CCGAATAGTTCGCCGAAAGAA

Abbreviation: qRT-PCR: quantitative real-time PCR.

## Data Availability

The data used to support the findings of this study are available from the corresponding author upon request.

## Supplementary Materials

Supplementary Materials supplementary Figure S1: the quantitative data of western blotting of relative LC3II/LC3I ratio.
