# Supplementary material for: Apigenin Alleviates Liver Fibrosis by Inhibiting Hepatic Stellate Cell Activation and Autophagy via TGF-β1/Smad3 and p38/PPARα Pathways
Source: PPAR Res. 2021 Jan 28;2021:6651839. doi: 10.1155/2021/6651839 (PMC7861947; doi:10.1155/2021/6651839)
Supplement: Supplementary Materials — supplementary Figure S1: the quantitative data of western blotting of relative LC3II/LC3I ratio. [file 6651839.f1.docx]

# Apigenin alleviates liver fibrosis by inhibiting hepatic stellate cell activation and autophagy via TGFβ1/smad3 and p38/PPARα pathways.

supplementary figure:

S1:


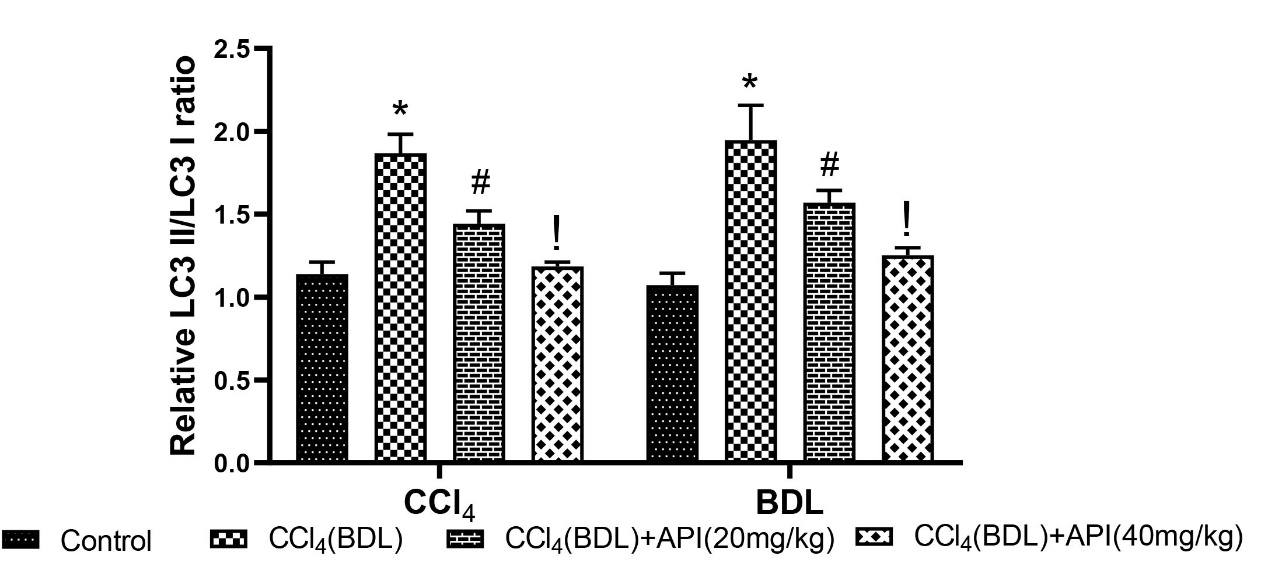


**S1**: the quantitative data of western blotting of relative LC3Ⅱ/LC3Ⅰ ratio

**Note:** n=6; **P*<0.05 for CCl_4_(BDL) vs Control; #*P*<0.05 for CCl_4_(BDL)+API (20mg/kg) vs CCl_4_(BDL); !*P*<0.05 for CCl_4_(BDL)+API(40mg/kg) vs CCl_4_(BDL)+API(20mg/kg).
